# Memory and Cognition-Related Neuroplasticity Enhancement by Transcranial Direct Current Stimulation in Rodents: A Systematic Review

**DOI:** 10.1155/2020/4795267

**Published:** 2020-02-25

**Authors:** Carla Cavaleiro, João Martins, Joana Gonçalves, Miguel Castelo-Branco

**Affiliations:** ^1^Coimbra Institute for Biomedical Imaging and Translational Research (CIBIT), University of Coimbra, Coimbra, Portugal; ^2^Institute of Nuclear Sciences Applied to Health (ICNAS), University of Coimbra, Coimbra, Portugal; ^3^CNC.IBILI Consortium, University of Coimbra, Coimbra, Portugal; ^4^Coimbra Institute for Clinical and Biomedical Research (iCBR), Faculty of Medicine, University of Coimbra, Coimbra, Portugal

## Abstract

Brain stimulation techniques, including transcranial direct current stimulation (tDCS), were identified as promising therapeutic tools to modulate synaptic plasticity abnormalities and minimize memory and learning deficits in many neuropsychiatric diseases. Here, we revised the effect of tDCS on the modulation of neuroplasticity and cognition in several animal disease models of brain diseases affecting plasticity and cognition. Studies included in this review were searched following the terms (“transcranial direct current stimulation”) AND (mice OR mouse OR animal) and according to the PRISMA statement requirements. Overall, the studies collected suggest that tDCS was able to modulate brain plasticity due to synaptic modifications within the stimulated area. Changes in plasticity-related mechanisms were achieved through induction of long-term potentiation (LTP) and upregulation of neuroplasticity-related proteins, such as *c-fos*, brain-derived neurotrophic factor (BDNF), or N-methyl-D-aspartate receptors (NMDARs). Taken into account all revised studies, tDCS is a safe, easy, and noninvasive brain stimulation technique, therapeutically reliable, and with promising potential to promote cognitive enhancement and neuroplasticity. Since the use of tDCS has increased as a novel therapeutic approach in humans, animal studies are important to better understand its mechanisms as well as to help improve the stimulation protocols and their potential role in different neuropathologies.

## 1. Introduction

**Figure 1:**
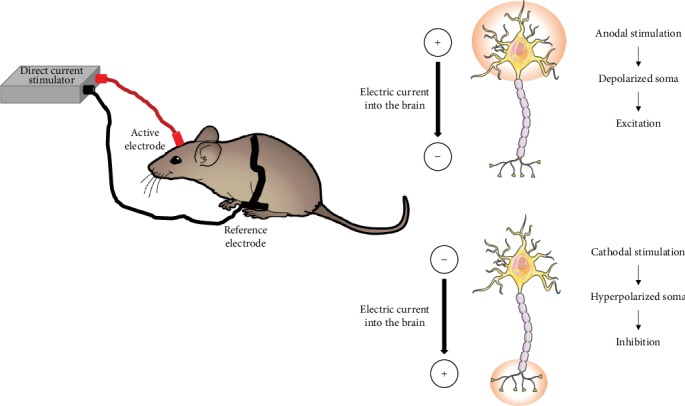
Illustration of transcranial direct current stimulation in the mice. Anodal stimulation depolarizes the neuronal membrane and enhances excitability. On the other hand, cathodal stimulation hyperpolarizes the neuronal membrane and decreases excitability.

Transcranial direct current stimulation (tDCS) is a noninvasive brain stimulation technique that promotes transient polarity-dependent changes in spontaneous neuronal activity. This effect is mediated by the application of constant low-amplitude electrical currents using epicranially positioned electrodes above a specific brain region of interest [1–4]. The therapeutic use of low-amplitude electrical currents has a long historical track. Accordingly, both Greeks and Romans used electric torpedo fishes for migraine treatment, and in the 11^th^ century, a similar therapeutic procedure was attempted to handle epilepsy [5]. In the 19^th^ century, the application of galvanic currents was attempted to heal melancholia [6]. Over the years, the scientific community interest in brain stimulation grew, and several noninvasive brain stimulation techniques were developed such as tDCS, deep brain stimulation, or transcranial magnetic stimulation. The epicranial application of direct currents promotes a weak electric field force and produces neuronal membrane potential changes [7, 8]. These alterations occur through sodium and calcium currents [1] modulating spontaneous neuronal activity [2]. The consequent regional neuronal inhibition or excitation depends on the applied current polarity [9, 10]. So, it was overall observed that cathodal currents produce inhibitory effects, and thus hyperpolarization, whereas anodal currents increase excitability in the form of depolarization [2, 11] (Figure 1).

There is nowadays an ongoing discussion regarding the factors that interfere with tDCS outcomes. The initial brain resting state of each subject [12], his/her baseline performance [13], specific individual variations in brain tissue morphology [14], or even more particular details from the experimental design or stimulation protocol used [15] influence these outcomes. *In vivo* and *in vitro* studies are consensual to demonstrate that tDCS-modulated cortical excitability depends on several stimulation parameters, such as duration and frequency of stimulation [16]; polarity, intensity, and density of the applied current [17, 18]; and electrode size and position in the scalp [18–20]. Despite that, beneficial effects of tDCS in several brain disorders, such as PD [21, 22], depression [23], stroke [24, 25], or autism [26, 27], have been documented, and there is growing evidence proposing tDCS application in multiple other disease conditions affecting cognition and neuroplasticity mechanisms.

Both preclinical and clinical studies have demonstrated therapeutic effects of tDCS. Indeed, in human studies, anodal tDCS applied intermittently in the prefrontal cortex (PFC) during slow-wave sleep period, improved recall of declarative memories (word pairs). The authors correlated these findings with enhancement of slow oscillatory electroencephalogram (EEG) activity (<3 Hz, delta (*δ*) waves), responsible for neuronal plasticity facilitation [28]. Also, anodal tDCS over dorsolateral prefrontal cortex (DLPFC) improved working memory in PD patients and in major depression patients by boosting cortical excitability [21, 23]. Accordingly, preclinical animal studies reported that cortical anodal tDCS improved spatial memory in both wild type (WT) [29] and the AD rat model [30]. Beneficial effects were also found during the early stage of traumatic brain injury (TBI) [31] and following a pilocarpine-induced *status epilepticus* in normal rats [32]. Moreover, improvements were also reported concerning short-term memory in an animal model of attention deficit hyperactivity disorder (ADHD) [33].

The molecular mechanisms underlying the tDCS-mediated cognitive improvements and neuroplasticity processes have become the focus of recent interest. Accordingly, tDCS modulation over several cognition-related plasticity genes and their signaling pathways has been studied. In this review, we provide a state of the art on the application of different protocols of tDCS in animal models highlighting its effectiveness on neuroplasticity mechanisms and, consequently, their related learning and memory processes. Since the published systematic reviews focused on human application of tDCS, here, we provide a comprehensive revision of the effect of tDCS in *in vivo* rodent models of normal and pathological brain functioning.

## 2. Methods

### 2.1. Data Sources and Search

Studies included in this review were identified by searching PubMed. The search was run until 31 October 2019. The search terms were (“transcranial direct current stimulation”) AND (mice OR mouse OR animal). Articles were firstly assessed based on their abstracts and titles, aiming to include studies that reported applying tDCS to cognitive impairment in animal models. Simultaneously, the following exclusion criteria were adopted to reject studies: (1) not written in English; (2) performing reviews; (3) in human subjects; (4) *in vitro* models; (5) employing other brain stimulation techniques (e.g., transcranial magnetic stimulation (TMS), deep brain stimulation (DBS), or transcranial alternating current stimulation (tACS)); and (6) not explicitly describing the tDCS protocol (stimulation area, number of sessions, frequency, intensity, and pattern).

### 2.2. Data Extraction

A data extraction sheet was developed seeking to retrieve relevant information from each study, namely, study design, sample size, animal model, whether additional therapy was performed, details of the tDCS protocol, outcome measures, and behavioral results.

### 2.3. Study Selection

**Figure 2:**
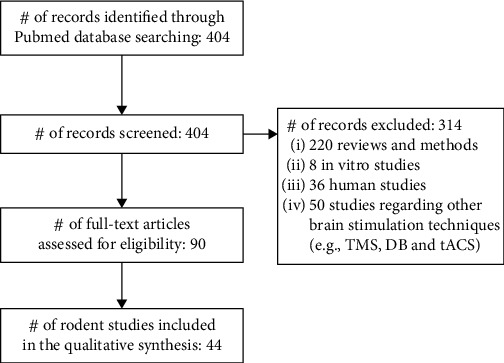
Search flow diagram (in accordance with PRISMA statement). Abbreviations: DB: deep brain stimulation; tACS: transcranial alternating current stimulation; TMS: transcranial magnetic stimulation.

The database search was elaborated according to the PRISMA statement requirements [34]. 404 records were found, which underwent a preliminary screening (of titles and abstracts), with 314 records being excluded because they did not meet the eligibility criteria. After the full-text analysis of each of the 90 individual articles, 44 rodent studies focusing on tDCS effects over cognition and neuroplasticity in both healthy and neuropathological animal models were selected (Figure 2).

## 3. Results

### 3.1. Role of Anodal tDCS in Cognition Processing in Healthy Animals

In healthy animals, studies demonstrated memory improvement in association with induction of synaptic plasticity mechanisms. In fact, tDCS to prefrontal cortex improved monkey's performance on an associative learning task by altering low-frequency oscillations and functional connectivity, both locally and between distant brain areas [35]. Regarding rodent models, data are controversial regarding fear condition. Right frontal anodal tDCS administered 24 h before behavioral task did not alter contextual and auditory learning and memory [36]. Additionally, another study described that while the anodal stimulation did not affect fear retrieval, posttraining cathodal stimulation improved fear memory retrieval [37, 38]. However, left prefrontal anodal and cathodal tDCS impaired the acquisition of both contextual and cued fear memory, which could be explained by activity modulation of deep structures such as the amygdala and hippocampus [39].

Concerning learning and memory, de Souza Custódio and colleagues [29] reported better spatial working memory performance following administration of anodal currents to the medial prefrontal cortex (mPFC). In agreement, it was described that administration of hippocampal anodal tDCS improves learning and memory in the Morris water maze and novel object recognition tests [40]. Moreover, memory performance in the passive avoidance learning task was enhanced by anodal stimulation [41]. Also, cortical cathodal stimulation together with visuospatial memory training led to cognitive improvement [42].

The revised *in vivo* animal model studies regarding tDCS effects in memory and cognition of healthy animals are listed below in Table 1.

### 3.2. Beneficial Role of tDCS in Brain Diseases

Overall, reports using animal models of brain diseases described a beneficial role of tDCS in the mitigation of memory symptoms of neurologic conditions such as Alzheimer's disease (AD) or traumatic brain injury (TBI). More recent studies demonstrated that tDCS rescued AD-related cognitive symptoms, namely, spatial memory and motor skills [30, 43, 44]. The repetitive stimulation with anodal tDCS in the AD-like dementia rat model reduced the time interval animals needed to reach a food pellet and also decreased the number of errors in the attempt [43]. The same research group showed later that the abovementioned protocol rescued spatial learning and memory in a A*β*_1-40_-lesioned AD rat model [30]. Moreover, the impact of tDCS on cognitive performance of streptozotocin-induced diabetic rats has been evaluated. Both anodal and cathodal stimulations in the prefrontal cortex restored memory impairment [45, 46] together with restoration of LTP [45]. Other authors evaluated the potential therapeutic effects of tDCS in memory impairment in an animal model of ADHD. It was found that this neuromodulation technique was able to improve short- and long-term memory deficits in the spontaneous hypertensive rats (SHR) but not in their control, Wistar Kyoto rats [33, 47]. In addition, no changes were detected in working memory of these control rats following administration of tDCS [47].

Anodal tDCS also ameliorated behavioral and spatial memory function in the early phase after TBI when it was delivered two weeks postinjury. However, earlier stimulation only improved spatial memory [31]. In a later phase of TBI, it was possible to observe motor recovery as well as spatial memory improvement following repeated anodal tDCS [48]. A growing number of studies has been reporting promising effects of neurostimulation in models of addictive disorders, by reducing craving and maladaptive pervasive learning [49]. In fact, repeated anodal stimulation in mouse frontal cortex decreased nicotine-induced conditioned place preference and further improved working memory [50]. Same polarity currents also could prevent cocaine-induced locomotor hyperactivity and place preference conditioning [51]. In addition, it has been reported that cathodal stimulation has an anticonvulsive effect [16, 32, 52–54]. Indeed, the administration of hippocampal tDCS rescued cognitive impairment by reducing hippocampal neural death and supragranular and CA3 mossy fiber sprouting in a *lithium*-pilocarpine-induced *status epilepticus* rat [32]. Other neuroplastic effects were evidenced in the reversion of motor symptoms in PD by tDCS administration. The application of anodal currents enhanced graft survival and dopaminergic re-innervation of the surrounding striatal tissue and pronounced behavioral recovery [55].

Despite the fact that many studies reported recovery from memory deficits following tDCS stimulation, there are some opposing reports in animal models of disease affecting cognition. In a recent study from Gondard and collaborators using a triple transgenic (3xTg) mouse model of AD, it was evidenced that a neurostimulation was not able to ameliorate memory symptoms [56]. To reconcile this discrepancy, previous authors have suggested the importance of choosing an optimal current intensity in order to modulate cortical excitability since LTP alterations were dependent on current intensity [57].

The reports regarding tDCS effects in cognition and memory in animal models of brain disease are listed in Tables 2 and 3.

### 3.3. Effect of tDCS on Cellular and Molecular Neuroplasticity Mechanisms

Neuronal network reorganization underlies neuroplasticity processes like developmental synaptogenesis, or neurogenesis and synaptic turnover later on, which ultimately contributes to optimal brain development and aging, as well as functional recovery upon trauma [58]. Interestingly, several reports using genetic engineered animals, pharmacologically induced animal models of disease, or *in vitro* techniques enlightened the potential of direct current stimulation (DCS) to interact with a myriad of neuroplasticity-related processes such as neuroinflammation [59, 60], neural stem cell migration [59], neurite growth [61], or neurogenesis [62]. Moreover, both human and *in vivo* animal studies evidenced a tDCS-induced effect on memory and learning [28, 35, 63]. However, the underlying cellular and molecular mechanisms remain to be elucidated.

#### 3.3.1. Modulation of the Excitatory/Inhibitory Network

To date, animal experimental evidence highlighted tDCS influences on synaptic plasticity, through alterations in the functional connectivity of cognition-related areas [35] and by modulation of excitatory/inhibitory network *tonus* [64], which may involve both the GABAergic and glutamatergic systems. Accordingly, a study conducted with older adults remarked an anodal stimulation effect in gamma-aminobutyric acid (GABA) levels [65]. Similarly, in human healthy volunteers, an anodal tDCS effect in motor learning was correlated with a decrease in GABA levels, an outcome known to be a determinant factor in the promotion of long-term potentiation- (LTP-) dependent plasticity and therefore learning [66, 67].

Several preclinical studies probed LTP enhancement following direct current stimulation. Anodal DCS enhanced LTP in both mouse cortex [68] and rat hippocampal slices [69, 70]. Further, this neurostimulation method increased local field potential (LFPs) amplitudes in primary somatosensory cortex of rabbits [63]. Also, other works demonstrated that neurostimulation-enhanced hippocampal LTP was associated with better spatial memory performance along with an increase in brain-derived neurotrophic factor (BDNF) expression levels [40]. An opposite effect on LTP and LFPs was obtained with administration of cathodal currents. In agreement, a report from Sun et al. [71] evidenced that cathodal currents applied in mouse neocortical slices induced field excitatory postsynaptic potential depression. This type of LTD was smothered by application of an mGluR5 negative allosteric modulator [72]. These findings support a possible modulatory effect of tDCS on mGluR5-mTOR signaling [72]; these molecular pathways are recognized to disturb cognition-related synaptic plasticity.

Further evidence supporting tDCS effect on LTP-like mechanisms was recently brought to light by Stafford et al. [73]. These authors observed that a single anodal tDCS increased both the phosphorylation at the S831 of GluA1 subunit and the translocation of *α*-amino-3-hydroxy5-methyl-4-isoxazole propionic acid receptors (AMPARs) from cytosolic to synaptic fractions in the hippocampus. These data could be favoring learning enhancement, as this translocation has been associated with hippocampal LTP induction [72]. Accordingly, others reported a spatial working memory enhancement after anodal stimulation over left medial PFC that was lost with the administration of the AMPAR antagonist perampanel (PRP). In contrast to cathodal currents, anodal currents enhanced intracellular calcium (Ca^2+^) intake in cell cultures including astrocytes [74–76], a process associated with AMPAR phosphorylation and trafficking to postsynaptic density [77] and ultimately, allowing LTP facilitation, a cellular correlate of learning and memory.

#### 3.3.2. Activation of Neuroplasticity-Associated Gene Expression

Neurostimulation could have long-lasting effects in memory as data from different studies evidenced [40]. Authors have been argued that tDCS cognition modulation is associated with neuroplasticity-associated gene expression alterations [78]. One of the neuroplasticity-associated genes, known to be essential for hippocampal LTP, is BDNF [79]. Several studies elucidated the role of BDNF in memory modulation by tDCS. In fact, it was reported that anodal currents could increase BDNF expression [68], and its activation *via* tropomyosin receptor kinase (Trk) receptors [80], triggering NMDAR opening, and inducing a later phase LTP (L-LTP) facilitation [81]. Accordingly, Yu et al. [41] found that the administration the Trk inhibitor ANA-12 prevented the anodal tDCS-induced hippocampal CA1 LTP increase. Other studies, using the same polarity currents, revealed a link between the upregulation of BDNF and cAMP response element binding protein/CREB-binding protein (CREB/CBP) [40] involved in LTP and memory formation [82]. Also, the application of cortical anodal currents in frontal cortex was able to upregulate BDNF together with striatal dopamine [33]. The upregulation of BDNF following neurostimulation was associated with the augmentation of expression levels of immediate early genes (IEGs), such as c-fos and zif268 [69]. Moreover, Kim et al. [78] confirmed that repetitive anodal tDCS in right sensorimotor cortex of healthy rats promoted a significant increase of mRNA levels of plasticity-associated genes, namely, BDNF, cAMP response element binding protein (CREB), synapsin I, Ca^2+^/calmodulin-dependent protein kinase II (CaMKII), activity-regulated cytoskeleton-associated protein (Arc), and c-fos. It was also demonstrated that sensory evoked cortical responses were boosted after tDCS *via* alpha-1 adrenergic receptor-mediated astrocytic Ca^2+^/IP3 signaling, thus involving also glia and the adrenergic system [75]. Anodal tDCS actions in glia were further confirmed by Mishima et al. [76]. Using a mouse model lacking Ca^2+^ uptake in astrocytes, the inositol trisphosphate receptor type 2 (IP3R2) knockout (KO) mouse and also an adrenergic receptor antagonist, they confirmed decreased microglia motility along with soma enlargement in tDCS stimulated animals [76].

In poststroke recovery, it was found that anodal currents significantly increased the GAP-43 and the microtubule-associated protein 2 (MAP-2) expression around the infarct area [56]. These neuronal growth-promoting proteins are overexpressed during dendritic remodeling and axonal regrowth throughout the acute phase of stroke [83, 84]. Anodal stimulation also modulated pannexin-1 (PX1) hemichannel levels [85, 86] and, following an ischemic insult, neurostimulation decreased rat PX1 mRNA and, consequently, augmented dendritic spine density in the surrounding areas of cerebral infarction; these cellular outcomes were associated with the improvement of motor function [85]. Some authors proposed that tDCS-induced improvement of stroke/TBI symptoms might be due to increase of BDNF expression and associated with choline/creatine ratios in the perilesional cortex [31].

**Figure 3:**
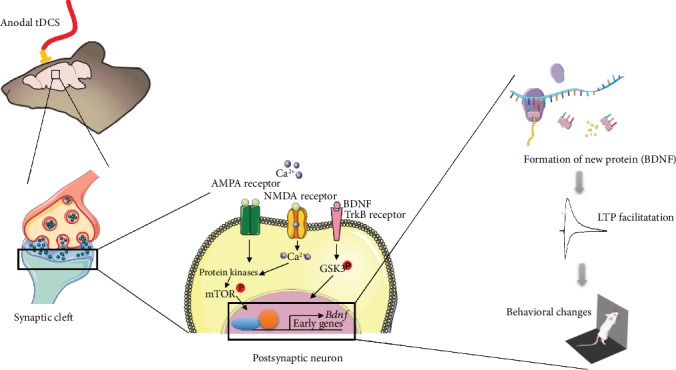
Schematic illustration of molecular mechanisms underlying the effect of anodal transcranial direct current stimulation (tDCS) on neuronal physiology. The neurostimulation in the target cortical area depolarizes neuronal membrane and glutamate released in presynaptic neuron and binds in NMDA and AMPA receptors (see book chapter Rozisky et al., 2015). Consequently, there is intracellular Ca^2+^ upregulation in the postsynaptic neuron, which can activate protein kinases that in turn modulate numerous neuronal signaling pathways (such as the mTOR pathway) leading to transcriptional changes. The tDCS also activates molecular cascades to promote BDNF production. As a long-term mechanism, gene transcription is modulated leading to the formation of new proteins that in turn lead to facilitation of LTP and improvement of cognition. Abbreviations: AMPA: *α*-amino-3-hydroxy-5-methyl-4-isoxazolepropionic acid; BDNF: brain-derived neurotrophic factor; CBP: CREB-binding protein; CREB: cAMP response element binding protein; GSK3: glycogen synthase kinase 3; LTP: long-term potentiation; mTOR: mammalian target of rapamycin; NMDA: N-methyl-D-aspartate; TrkB: tropomyosin receptor kinase B.

Overall, tDCS methodology was able to modulate molecular pathways involved in the regulation of cognition-related synaptic plasticity mechanisms (Figure 3). The revised *in vivo* animal studies regarding tDCS-induced effects in the cellular and molecular mechanisms of memory and learning are listed in Table 4.

## 4. Discussion

This systematic review collected several studies that confirm the potential effects of tDCS on neuronal activity and synaptic plasticity. Here, we documented a variable combination of stimulation protocols, stimulation areas, and healthy and disease animal models. Most of the existent literature is focused on human application of tDCS. The comprehensive revision of the effect of tDCS on rodent models of normal and pathological brain functioning does therefore provide a novel contribution to the field. Overall, the revised studies indicated that tDCS was able to modulate synaptic plasticity and, consequently, learning and memory processes [87, 88].

Memory formation and consolidation are recognized to rely on activity-dependent modifications, such as LTD and LTP [89], both dependent on the activation of calcium-dependent kinases (e.g., CaMKs), which in turn control the trafficking of NMDARs and AMPARs [90]. Despite the wide set of stimulation protocols, tDCS-induced modulation of NMDAR signaling and synaptic protein upregulation resulting in LTP and cognitive enhancement have been consistently reported in animal studies. Anodal tDCS increased AMPAR synapse translocation [73, 89] and induced spatial memory improvement by involving both CREB and BDNF expression alterations [53]. Also, an increase in hippocampal and cortical mRNA levels of c-fos, synapsin, CaMKII, and Arc was observed poststimulation [78].

Similar results highlighting tDCS effects in neuroplasticity were obtained with *in vitro* studies. Accordingly, Ranieri and coworkers [69] probed that anodal currents increased NMDAR-dependent LTP in hippocampal CA3-CA1 synapses [69], in part, due to production of BDNF [68]. In addition, it was demonstrated that tDCS-induced hippocampal BDNF release is dependent on histone acetylation of BDNF gene promoters [40]. Overall, the abovementioned works provide positive evidence for the effect of tDCS on cognitive function enhancement.

Although tDCS impaired the acquisition of both contextual and cued fear memory [39], there are no studies on possible cascades/proteins involved in tDCS-induced neuroplasticity alterations following fear memory changes. Nevertheless, a very recent paper demonstrated chronic repetitive TMS of the ventromedial prefrontal cortex reversed stress-induced behavior impairments with an increase of c-fos activity [91].

Cortical anodal currents have been shown to be mostly excitatory and support memory enhancement and neuroplasticity. The literature is also consistent with the notion that the stimulation over the cortical region functionally involved in a certain cognitive task increases performance in that specific task. Marshall et al. demonstrated that anodal currents over the PFC, a region involved in memory encoding, during slow wave sleep improved declarative memory [28]. However, it was described that cortical cathodal stimulation simultaneously with training task was able to increase visuospatial working memory, in spite of the fact that it was associated with decreased excitability [42]. This suggests that modulatory effects of tDCS were influenced by the polarity-dependent electrical dynamics established between the stimulated area and its related neuronal networks. In agreement, a recent report observed an inhibitory effect in motor learning tasks following anodal currents in the cerebellum; the anodal excitatory effect over the Purkinje cell activity led to an overall inhibition of downstream structures, reducing as a result the vestibulo-ocular reflex gain [90]. Similar paradoxical results have been observed in humans. Recently, Moliadze and collaborators [92] reported that tDCS-induced neural modulation depended on several parameters, namely, the age. In fact, an excitatory effect was seen in young subjects, but not in the older participants.

Nowadays, TMS, another important noninvasive brain stimulation technique, is useful for evaluating excitability in the primary motor cortex (M1) and conductivity along the cortical-spinal tract. This technique has been amply used in rehabilitation of stroke patients [93] and in neuropsychiatric disorders, namely, depression [94]. tDCS and TMS are undergoing the most active investigation and share a capacity to modulate regional cortical excitability, and both are well-tolerated by children and adults [95]. However, TMS has been already approved for clinical use and tDCS is still undergoing investigation as a plausible therapy for a range of neuropsychiatric disorders [95]. The rational, in part, for this is because data on the efficacy and safety of tDCS are sparse and employ heterogeneous stimulation protocols. Indeed, there is a paucity of strictly conducted randomized, sham controlled clinical trials, and case considerable follow-up periods, which makes it difficult to use these results to inform clinical practice concerning the putative beneficial role of tDCS. Moreover, tDCS effects seem to be clearly dependent on structure, connectivity, and function of the target brain region. Importantly, these outcomes were intrinsically correlated with GABAergic neurotransmission which raises the issue that one has to take into account that during development GABA can act as an excitatory neurotransmitter [96].

## 5. Conclusions

There is growing evidence that tDCS modulates brain activity and, consequently, enhances synaptic plasticity and cognitive performance. Overall, reports from laboratory animal research present tDCS as a promising noninvasive brain stimulation technique. The presented evidence is therefore consistent with human studies suggesting that this technique is useful to mitigate neurologic symptoms of several brain disorders, thus improving learning and memory. Further research is needed so that this technique can be fully translated into optimal therapeutic strategies.

## Figures and Tables

**Table 1:** Effect of transcranial direct current stimulation on memory and learning of healthy animals.

Author	Year	Animal model	Specimen; gender	*N*	Stimulation parameters										Main findings
					Stimulation electrode						Reference electrode		Anesthesia	rtDCS	
					Polarity	Position	Stimulation intensity (mA)	Size (m^2^)	Stimulation duration (min)	Current density (A/m^2^)	Position	Area (cm^2^)			
Dockery et al. [42]	2011	NDM	Long-Evans rats; males	41	a-tDCS vs. c-tDCS	Frontal cortex (left or right hemisphere)	0.2	0.035	30	57.1	Back	10.5	N	Y (3 days)	↑ Visuospatial working memory (c-tDCS)

de Souza Custódio et al. [29]	2013	NDM	Wistar rats; males	23	a-tDCS	Left mPFC	0.4	0.25	11	N/A	Neck	1	N	Y (5 days)	↑ Spatial working memory (1 h, 4 h, and 10 h poststimulation)

Faraji et al. [96]	2013	NDM	Long-Evans rats; males	24	a-tDCS	Somatosensory cortex (bilateral)	0.065	N/A	10	N/A	Back of skull	N/A	N	Y	↑ Cortical neural density ↑ Motor learning (a-tDCS applied bilaterally or into paw preferred to reaching contralateral hemisphere)

Podda et al. [40]	2016	NDM	C57BL/6 mice; males	16	a-tDCS vs. c-tDCS	Left parietal cortex (dorsal to hippocampal formation)	0.35	0.06	20	N/A	Ventral thorax	5.2	N	N (single session)	↑ Spatial learning and memory (a-tDCS; benefits observable one week after) ↑ BDNF expressions in the *hippocampus* CREB/CBP pathway activation

Manteghi et al. [36]	2017	NDM	NMRI mice; males	64	a-tDCS	Right frontal cortex	0.2	0.04	20	N/A	Chest	9.5	N/A	N (single session)	↓ Freezing time % and ↑ latency to the freezing (tDCS following 0.1 mg/kg ACPA injection)

Nasehi et al. [37]	2017	NDM	NMRI mice; males	128	a-tDCS vs. c-tDCS	Right frontal cortex	0.2	0.04	20	N/A	Ventral thorax	9.5	N	Y (2 sessions)	↑ Fear memory retrieval/freezing time (a-tDCS and propranolol injection before conditioning)

Nasehi et al. [38]	2017	NDM	NMRI mice; males	128	a-tDCS vs. c-tDCS	Left frontal cortex	0.2	0.04	20	N/A	Ventral thorax	9.5	N	Y (2 sessions)	↑ Contextual fear memory acquisition (a-tDCS before pre- or posttraining)

Abbasi et al. [39]	2017	NDM	NMRI mice; males	41	a-tDCS vs. c-tDCS	Left PFC	0.2	0.04	20 or 30	N/A	Chest	9.5	N	N (single session)	↓ Contextual and cued fear memory

Martins et al. [97]	2019	NDM	Male Wistar rats; males	50	a-tDCS	Left mPFC	0.4	N/A	13	N/A	N/A	N/A	N/A	Y (5 days)	↑ Spatial working memory ↑ GAP-43 (extinct by AMPAR antagonist PRP)

Yu et al. [41]	2019	NDM	Sprague Dawley rats; males	224	a-tDCS	SC dorsal to the *hippocampus*	0.25	0.25	30	N/A	Anterior chest	N/A (EEG electrode)	Y	N (single session)	↑ Memory (passive avoidance memory retention) ↑ LTP in CA1 *hippocampus* (blocked by TrkB antagonist) ↑ BDNF in CA1 *hippocampus*

Abbreviations: rtDCS: repetitive transcranial direct current stimulation; a-tDCS: anodal transcranial direct current stimulation; c-tDCS: cathodal transcranial direct current stimulation; SC: stereotaxic coordinates; C57BL/6: mouse strain; NMRI: Naval Medical Research Institute outbred mice; NBM: no disease model; SHR: spontaneous hypertensive rats; WKY: Wistar Kyoto rats; PFC: prefrontal cortex; mPFC: medial prefrontal cortex; ITC: inferotemporal cortex; CAI: comu ammonis 1 region in the hippocampus; PRP: perampanel; ACPA: anticitrullinated protein antibody (selective cannabinoid CBI receptor agonist); AMPAR: *α*-amino-3-hydroxy-5-methyl-4-isoxazolepropionic acid receptor; Trk: tropomyosin receptor kinase receptor; CREB/CBP: cAMP response element binding protein; BDNF: brain-derived neurotrophic factor; GAP-43: growth-associated protein 43; LTP: long-term potentiation; EEG: electroencephalography; A/m^2^: ampere per square meter; mA: milliampere; cm^2^: square centimeter; mm: millimeter; h: hour; min: minute; vs.: versus; Y: yes; N: no; N/A: not available.

**Table 2:** Impact of transcranial direct current stimulation on memory and learning in animal models of brain disorders.

Author	Year	Animal model	Specimen; gender	*N*	Stimulation parameters										Main findings
					Stimulation electrode						Reference electrode		Anesthesia	rtDCS	
					Polarity	Position	Stimulation intensity (mA)	Size (cm^2^)	Stimulation duration (min)	Current density (A/m^2^)	Position	Area (cm^2^)			
Kamida et al. [32]	2011	Lithium-pilocarpine hydrochloride (60 mg/kg) SC injection at P20-21	Wistar rats; males	18	c-tDCS	Hippocampus	0.2	0.035	30	N/A	Back of neck	N/A	N	Y (daily for 2 weeks)	↓ SE-induced hippocampal cell loss in CA3 region

Yu et al. [43]	2014	Scopolamine IP injection	Sprague Dawley rats; both genders	16	N/A	Parietal cortex (hippocampus)	0.1	N/A	20	N/A	N/A	N/A	N/A	Y (2x/day during 5 days a week, for 4 weeks)	↓ Time and ↓ number of errors to reach food pellets Significant differences in ACh content (day 14 and day 28) ↑ Motor function

Pedron et al. [49]	2014	Nicotine IP injection (1 mh/kg 2x/day for 14 days)	Swiss mice; females	152	a-tDCS	Left frontal cortex	0.2	0.035	2 × 20	N/A	Ventral thorax	9.5	N	Y (5 days)	↑ Working memory
															↑ Nicotine-induced place preference conditioning (3 weeks poststimulation)

Yu et al. [30]	2015	Bilateral *hippocampus* A*β*_1-40_ injection	Sprague Dawley rats; females	36	a-tDCS	Right frontal cortex	0.02; 0.06; 0.1; 0.2	0.0314	20	N/A	Ventral thorax	10	N	Y (10 sessions in 2 weeks)	↑ Spatial learning performance (best with 0.1 mA and 0.2 mA stimulation)
															↓ GFAP expression in CA1 *hippocampus* region and DG (best with 0.1 mA stimulation)

Yoon et al. [31]	2016	Lateral fluid percussion method	Sprague Dawley rats; males	36	a-tDCS	Hippocampus	0.2	0.0225	20	28.2	Chest	48 (corset)	Y	Y (daily for 5 days)	↑ Perilesional area BDNF expression (tDCS 2 weeks post-TBI) ↑ Choline/creatinine ratios (tDCS 1 week post-TBI) Motor performance recovery (2 weeks of tDCS)

Leffa et al. [33]	2016	SHR	SHR rats and WKT rats; males	48	a-tDCS	Frontal cortex	0.5	1.5	20	33.4	Between ears	1.5	N	Y (8 consecutive days)	↑ DA levels in STR in both rat strains and in the hippocampus following tDCS treatment in WKY ↑ BDNF levels in WKY rats Short-term memory improvement

Wu et al. [45]	2017	STZ-induced diabetic rats	Sprague Dawley rats; males	130	a-tDCS	dPFC	0.2	0.0314	30	N/A	Anterior chest	0.25	Y	Y	↑ Spatial working memory and mPFC LTP restoring

Pedron et al. [50]	2017	Cocaine injections	Swiss mice; females	165	a-tDCS	Left frontal cortex	0.2	0.035	2 × 20	N/A	Ventral thorax	9.5	N	Y (5 days stimulation, twice a day; 5 h interstimulation interval)	↓ Cocaine-induced locomotor activity No cocaine-induced place preference (5 mg/kg and 25 mg/kg) ↑ Zif268 basal expression under the electrode area, in the STR and cortex

Leffa et al. [55]	2018	ADHD	SHR and WKY rats; males	30	a-tDCS (bicephalic)	Frontal cortex (supraorbital area)	0.5	1.5 (ECG electrode)	20	33.3	Neck	1.5 (ECG electrode)	N/A	Y (8 days)	↓ Inflammatory cytokines and reversion of long-term memory deficits in SHR rats

Bragina et al. [47]	2018	CCI	Mice; N/A	40	a-tDCS	Parietal somatosensory cortex	0.1	N/A	15	N/A	Ventral thorax	N/A	N/A	Y (daily for 4 days, over 4 weeks and 3 days interval)	Motor coordination recovery Spatial memory and learning performance improvement ↑ CBF bilaterally (regional arteriolar dilatation and hypoxia reduction)

Roostaei et al. [44]	2019	STZ-induced diabetic rats	Wistar rats; males	64	a-tDCS vs. c-tDCS	Left frontal cortex	0.2	3.5	20	N/A	Ventral thorax	9.5	N	Y (twice a day over 2 days)	Restoration of STZ-induced amnesia (both polarities)

Gondard et al. [55]	2019	Animal model of AD-triple transgenic (3xTg) mice	Triple transgenic (3xTg) mice; males	27	c-tDCS and a-tDCS	Secondary motor cortex (m2) (c-TDCS) and dorsal temporal *hippocampus* (a-tDCS)	0.05	0.0325	20	N/A	N/A	0.0325	N/A	Y (5 days/week for 3 weeks)	No treatment effect on memory outcome or AD neuropathological biomarkers

Abbreviations: rtDCS: repetitive transcranial direct current stimulation; a-tDCS: anodal transcranial direct current stimulation; c-tDCS: cathodal transcranial direct current stimulation; TBI: traumatic brain injury; ADHD: attention deficit hyperactivity disorder; SHR: spontaneous hypertensive rats; WKY: Wistar Kyoto rats; CBF: cerebral blood flow; PFC: prefrontal cortex; mPFC: medial prefrontal cortex; dPFC: dorsolateral prefrontal cortex; DG: dentate gyrus; STR: striatum; M2: secondary motor cortex; ITC: inferotemporal cortex; CA3: cornu ammonis 3 region in the *hippocampus,* STZ: streptozotocin; AD: Alzheimer's disease; A*β*_1-40_: amyloid beta peptide 1–40; ACh: acetylcholine; BDNF: brain-derived neurotrophic factor; DA: dopamine; GFAP glial fibrillary acidic protein; Zif268: zinc finger transcription factor 268; LTP: long-term potentiation; IP: intraperitoneal; SC: subcutaneous; SE: *status epilepticus*; ECG: electrocardiography; mg: milligram; kg: kilogram; A/m^2^: ampere per square meter; mA: milliampere; cm^2^: square centimeter; mm: millimeter; h: hour; min: minute; vs.: versus; Y: yes; N: no; N/A: not available.

**Table 3:** Role of transcranial direct current stimulation on neuroplasticity with a focus on animal models of neurotrauma.

Author	Year	Animal model	Specimen; gender	*N*	Stimulation parameters										Main findings
					Stimulation electrode						Reference electrode		Anesthesia	rtDCS	
					Polarity	Position	Stimulation intensity (mA)	Size (cm^2^)	Stimulation duration (min)	Current density (A/m^2^)	Position	Area (cm^2^)			
Nekhendzy et al. [97]	2004	Inflammatory nociception model	Sprague Dawley rats; males	31	c-tDCS	Frontal cortex	2.25	N/A	45	N/A	Bimastoid	N/A	Y	Y (8 days)	↓ Nociceptive response (effects lasted up to 50 min poststimulation)

Taib et al. [98]	2009	Left hemicerebellectomy	Wistar rats; males	9	a-tDCS	Right or left motor cortex	0.4	0.071	20	56.3	Supraorbital region	0.0064	N/A	Y	↑ T corticomuscular response amplitudes

Kim et al. [99]	2010	MCAO	Sprague Dawley rats; both genders	61	c-tDCS vs. a-tDCS	Left primary motor cortex (M1)	0.1	0.785	30	N/A	Trunk	9	Y	Y (2 weeks)	Neuroprotection over white matter and ischemic size ↓ (a-tDCS)
															↑ Motor function

Jiang et al. [95]	2012	MCAO	Sprague Dawley rats; both genders	90	a-tDCS and c-tDCS	Motor cortex	0.1	0.785	30	1.27	Trunk	9	Y	Y (daily, for 3, 7, or 14 days postlesion)	↑ Motor function (7 and 14 days poststroke) ↑ Dendritic spine density ↓ PX1 expression (7th and 14 h day poststroke)

Yoon et al. [56]	2012	MCAO	Sprague Dawley rats; males	30	a-tDCS	Left primary motor cortex (M1)	0.2	N/A	20	28.2	Anterior chest	48.0	Y	Y (5 days stimulation 1 week vs. 1 day postischemia)	↑ Motor function and Barnes maze performance (a-tDCS applied 1 week after ischemic injury)
															↑ MAP-2 and GAP-43 expression around the perilesional area (a-tDCS applied 1 week postischemic injury)

Laste et al. [100]	2012	CFA injection/chronic inflammation induction	Wistar rats; males	18	a-tDCS	Parietal cortex	0.5	1.5 (ECG electrode)	20	33.4	Supraorbital area	1.5 (ECG electrode)	N	Y (8 days)	Significant differences in nociceptive response (immediately after and 24 h poststimulation)

Adachi et al. [101]	2012	CRS	Wistar rats; males	48	a-tDCS	Parietal cortex	0.5	1.5 (ECG electrode)	20	3.3	Supraorbital area	1.5 (ECG electrode)	N	Y (8 days)	↓ Nociceptive response in chronic stress condition ↓ TNF expression in the *hippocampus*

Peruzzotti-Jametti et al. [60]	2013	MCAO	C57BL/6 mice; males	137	c-tDCS vs. a-tDCS	Left parietal cortex	0.25	0.0144	40	55	Ventral thorax	5.2	N/A	N (single session)	↑ Infarct volume and ↑ BBB leakage (a-tDCS ipsilesional hemisphere)
															Cortical Glu activity ↓, functional ↑ and ischemic damage ↓ (c-tDCS ipsilesional hemisphere)

Notturno et al. [102]	2014	MCAO	Sprague Dawley rats; both genders	53	c-tDCS	Left motor cortex	0.2	0.07	120	28.6	Ventral thorax	10.5	Y	N (single session)	Ischemia volume ↓

Lu et al. [103]	2015	MPTP injection	C57bl mice; males	36	a-tDCS	Left frontal cortex	0.2	0.035	10	57	Between shoulders	9	N/A	Y (daily for 3 weeks)	↑ Motor coordination (until 21 days poststimulation) ↑ TH and DA expression ↓ Oxidative stress

Spezia Adachi et al. [104]	2015	Restraint stress model	Wistar rats; males	78	a-tDCS	Parietal cortex (midline)	0.5	1.5 (ECG electrode)	20	N/A	Supraorbital area	1.5 (ECG electrode)	N	Y (daily for 8 days)	↓ Stress-induced nociceptive response ↑ Pain threshold ↓ BDNF levels (spinal cord and brainstem) in unstressed animals

Yoon et al. [31]	2016	Lateral fluid percussion method	Sprague Dawley rats; males	36	a-tDCS	Hippocampus	0.2	0.0225	20	28.2	Chest	48 (corset)	Y	Y (daily for 5 days)	↑ Perilesional area BDNF expression (tDCS 2 weeks post-TBI)
															↑ Choline/creatinine ratios (tDCS 1 week post-TBI)
															Motor performance recovery (2 weeks of tDCS)

Leffa et al. [33]	2016	SHR	SHR rats and WKT rats; males	48	a-tDCS	Frontal cortex	0.5	1.5	20	33.4	Between ears	1.5	N	Y (8 consecutive days)	↑ DA levels in STR in both rat strains and in the hippocampus following tDCS treatment in WKY ↑ BDNF levels in WKY rats

Liu et al. [105]	2016	PTI	Sprague Dawley rats; N/A	58	c-tDCS	Right S1FL	2	N/A	20	20.37	Venter	N/A	N/A	N (single session)	Ischemia expansion inhibition (c-tDCS immediately postischemia induction) ↓ NeuN expression (c-tDCS+PSS group)

Braun et al. [106]	2016	MCAO	Wistar rats; males	41	c-tDCS vs. a-tDCS	Left primary motor cortex (M1)	0.5	0.035	15	N/A	Ventral thorax	N/A	Y	Y (10-day stimulation; 5 days with 2-day interval)	Gait recovery at day 16 poststroke (both polarities) Faster recovery of limb strength (fully recovered strength at day 10 and gait at day 14 with c-tDCS) ↑ Microglia and neuroblasts in lesion ipsilateral cortex

Cioato et al. [107]	2016	Sciatic nerve chronic constriction	Wistar rats; males	84	a-tDCS and c-tDCS	Parietal cortex (bicephalic)	0.5	1.5 (ECG electrode)	20	N/A	Supraorbital area	1.5 (ECG electrode)	N	Y (8 days)	Nociceptive relieve (for up to 7 days poststimulation) Reversion of T IL-1þ levels (48 h and 7 days poststimulation)

Filho et al. [108]	2016	Partial sciatic nerve compression	Wistar rats; males	144	a-tDCS	Parietal cortex	0.5	1.5 (ECG electrode)	20	N/A	Supraorbital area	1.5 (ECG electrode)	N	Y (8 days)	↓ BDNF expression (48 h poststimulation) Reversion of behavioral alterations (analgesic and anxiolytic) associated with neuropathic pain

Moreira et al. [109]	2016	Pain and menopause (ovariectomised animals)	Wistar rats; females	45	C-tDCS	Parietal cortex	0.5	1.5 (ECG electrode)	20	N/A	Supraorbital area	1.5 (ECG electrode)	N	Y (8 days)	↓ Hypothalamic BDNF levels and T serum BDNF in ovariectomised animals

Dimov et al. [87]	2016	N/A	Wistar rats; males	25	c-tDCS	Left primary motor cortex (M1)	0.25	0.0227	15	N/A	Ventral thorax	N/A	N	N (single session)	Bilateral ↓ Egr-1 expression in the PAG
															Spinal ENK immunoreactivity ↓ in the DHSC

Liu et al. [110]	2017	PTI	Sprague Dawley rats; males	58	c-tDCS	S1FL	2	N/A	20	N/A	3 mm lateral to lambda	N/A	N/A	N (single session)	Prevention of ischemia injury expansion during hyperacute phase of ischemia (c-tDCS+PSS)

Kim & Han [111]	2017	Modified Tang's method [128]	Sprague Dawley rats; N/A	31	a-tDCS	Left motor cortex	0.2	1	30	0.26	Ventral thorax	9	Y	N	Early recovery of consciousness and MEP and SEP prolonged latency (tDCS applied right after TBI) ↓ Astroglial GFAP immunoreactivity

Winkler et al. [112]	2017	Striatal 6-OHDA injection	Sprague Dawley rats; females	24	a-tDCS and c-tDCS	Left motor cortex	N/A	0.16	20	8	Chest	3	N	Y (daily for 14 days)	Graft survival, striatal dopaminergic reinnervation and motor recovery (a-tDCS)

de Souza et al. [113]	2017	PSNL	Swiss mice; males	N/A	a-tDCS and c-tDCS	Parietal cortex (bicephalic)	0.5	N/A (EEG electrode)	5; 10; 15; 20	N/A	Supraorbital area	N/A (EEG electrode)	N	N (single session)	Antiallodynic effect (seen 4 h poststimulation of 15 min and 20 min)

Leffa et al. [46]	2018	ADHD	SHR and WKY rats; males	30	a-tDCS (bicephalic)	Frontal cortex (supraorbital area)	0.5	1.5 (ECG electrode)	20	33.3	Neck	1.5 (ECG electrode)	N/A	Y (8 days)	↓ Inflammatory cytokines and reversion of long-term memory deficits in SHR rats

Paciello et al. [114]	2018	NIHL	Wistar rats; males	124	a-tDCS	Temporal lobe (auditory cortex)	0.35	0.0625	20	56	Ventral thorax	12	N	Y (2 days)	↑ Dendritic spines density (layer 2/3 pyramidal neurons of the auditory cortex)
															↑ BDNF and synaptophysin expression in auditory cortex (24 h poststimulation)

Fregni et al. [115]	2018	N/A	Wistar rats; males	32	N/A	N/A (bicephalic)	N/A	N/A	20	N/A	N/A	N/A	N/A	Y (8 days)	tDCS prior to stress exposure prevented thermal hyperalgesia

Lee et al. [116]	2019	MPTP injection	C57bl mice; male	60	a-tDCS	Primary motor cortex (M1)	N/A	N/A	30	N/A	Between shoulders	N/A	N/A	Y (daily for 5 days)	↑ Motor coordination
															Rescue of MTPT-induced mitochondrial dysfunction (Ç ATP and GDH and $ Drp1 levels)

Callai et al. [117]	2019	CCI-ION	Wistar rats, males	151	a-tDCS	Parietal cortex (bicephalic)	0.5	1.5 (ECG electrode)	20	N/A	Supraorbital area	1.5 (ECG electrode)	N	Y (8 days)	↓ Mechanical hyperalgesia
															↓ TNF-a expression (7 days poststimulation)
															↓ IL-10 (7 days poststimulation)
															↓ LDH serum levels

Scarabelot et al. [118]	2019	CFA injection/chronic inflammation induction	Sprague Dawley rats; males	104	a-tDCS	Parietal cortex (bicephalic)	0.5	1.5 (ECG electrode)	20	N/A	Supraorbital area	1.5 (ECG electrode)	N/A	Y (8 days)	↓ Thermal and mechanical hyperalgesia
															↑ IL-6 (in brainstem 24 h poststimulation)
															↓ IL-10 (7 days poststimulation)
															Normalization of BDNF levels (24 h poststimulation)

Abbreviations: rtDCS: repetitive transcranial direct current stimulation; a-tDCS: anodal transcranial direct current stimulation; c-tDCS: cathodal transcranial direct current stimulation; C57BL/6: mouse strain; SHR: spontaneous hypertensive rats; WKY: Wistar Kyoto rats; ADHD: attention deficit hyperactivity disorder; 6-OHDA: 6-hydroxydopamine; MPTP: 1-methyl-4-phenyl-1,2,3,6-tetrahydropyridine; TBI: traumatic brain injury; PTI: photothrombic ischemia; MCAO: middle cerebral artery occlusion; PSS: peripheral sensory stimulation; NIHL: noise-induced hearing loss; CC-ION: chronic constriction of the infraorbital nerve (pain model); PSNL: partial sciatic nerve ligation (pain model); CRS: chronic restraint stress (pain model); CFA: complete Freund's adjuvant (pain model); BBB: blood–brain barrier; S1FL: forelimb region of the primary somatosensory cortex; M1: primary motor area; PAG: periaqueductal grey; DHSC: dorsal horn of the spinal cord; MEP: motor-evoked potentials; SEP: somatosensory evoked potentials; GFAP: glial fibrillary acidic protein; BDNF: brain-derived neurotrophic factor; Glu: glutamate NeuN: neuronal marker; PX1: pannexin 1; TH: thyroxine hydroxylase; Egr-1: early growth response protein 1; TNF: tumor necrosis factor; IL-1þ: interleukin 1 beta; IL-6: interleukin 6; IL-10: interleukin 10; LDH: lactate dehydrogenase enzyme; ATP: adenosine triphosphate; GDH: glutamate dehydrogenase; Drp1: dynamin-related protein; MAP-2: microtubule associated protein 2; GAP-43: growth associated protein 43; ENK: early embryo specific NK; DA: dopamine; ECG: electrocardiography; EEG: electroencephalogram; A/m^2^: ampere per square meter; mA: milliampere; cm^2^: square centimeter; min: minute; vs.: versus; Y: yes; N: no; N/A: not available.

**Table 4:** Cellular and molecular mechanisms underlying transcranial direct current stimulation effect in the brain.

Author	Year	Animal model	Specimen; gender	*N*	Stimulation parameters										Main findings
					Stimulation electrode						Reference electrode		Anesthesia	rtDCS	
					Polarity	Position	Stimulation intensity (mA)	Size (cm^2^)	Stimulation duration (min)	Current density (A/m^2^)	Position	Area (cm^2^)			
Márquez-Ruiz et. [63]	2012	NDM	New Zealand White albino rabbits; N/A	13	a-tDCS and c-tDCS	Somatosensory cortex (S1)	0.5; 1; 1.5 and 2	0.7857	10	3.7	Ear	35	N	N (single session)	↑ LFP in S1 (a-tDCS) and ↓ LFP S1 (c-tDCS)

Rohan et al. [57]	2015	NDM	Sprague Dawley rats; male	34	a-tDCS	SC dorsal to the *hippocampus*	0.1 or 0.25	0.25	30	N/A	Between shoulders	8.04	N	N (single session)	# LTP and PPF in the *hippocampus*

Yoon et al. [31]	2016	Lateral fluid percussion method	Sprague Dawley rats; males	36	a-tDCS	*Hippocampus*	0.2	0.0225	20	28.2	Chest	48 (corset)	Y	Y (daily for 5 days)	# perilesional area BDNF expression (tDCS 2 weeks post-TBI) # choline/creatinine ratios (tDCS 1 week post-TBI) Motor performance recovery (2 weeks of tDCS)

Leffa et al. [33]	2016	SHR	SHR rats and WKT rats; males	48	a-tDCS	Frontal cortex	0.5	1.5	20	33.4	Between ears	1.5	N	Y (8 consecutive days	# DA levels in STR in both rat strains and in the hippocampus following tDCS treatment in WKY # BDNF levels in WKY rats Short-term memory improvement

Podda et al. [40]	2016	NDM	C57BL/6 mice; males	16	a-tDCS vs. c-tDCS	Left parietal cortex (dorsal to hippocampal formation)	0.35	0.06	20	N/A	Ventral thorax	5.2	N	N (single session)	# spatial learning and memory (a-tDCS); benefits observable one week after # BDNF expression in the *hippocampus* CREB/CBP pathway activation

Monai et al. [75]	2016	NDM	G7NG817 mice; N/A	10	a-tDCS	Primary visual cortex (VI)	0.1	0.02	10	N/A	Neck	N/A	N	N (single session)	Up to 50% expansion of visual evoke active area (up to 2 h poststimulation effect) tDCS-induced plasticity depends on the activity of IP, R2, and A1AR

Kim et al. [78]	2017	NDM	Sprague Dawley rats; males	90	a-tDCS	Right sensorimotor cortex	0.25	0.071	20	N/A	Right anterior chest	0.5	N	Y (7 days)	# BDNF, CREB, synapsin, and CaMKII mRNA expression levels (ipsilateral cortex) and c-fos (*hippocampus*)

Stafford et al. [73]	2018	NDM	Sprague Dawley rats, males	16	a-tDCS	Caudal to bregma	0.25	0.25	30	N/A	Ventral thorax	N/A	N	N (single session)	↑ AMPAR translocation to the synapse in the hippocampus and ↑ phosphorylation of the S831 site on GluA1

Martins et al. [97]	2019	NDM	Male Wistar rats; males	50	a-tDCS	Left mPFC	0.4	N/A	13	N/A	N/A	N/A	N/A	Y (5 days)	↑ Spatial working memory
															↑ GAP-43 (extinct by AMPAR antagonist PRP)

Yu et al. [41]	2019	NDM	Sprague Dawley rats; males	224	a-tDCS	SC dorsal to the *hippocampus*	0.25	0.25	30	N/A	Anterior chest	N/A (EEG electrode)	Y	N (single session)	↑ Memory (passive avoidance memory retention)
															↑ LTP in CA1 *hippocampus (blocked by TrkB antagonist)* ↑BDNF in CA1 *hippocampus*

Abbreviations: rtDCS: repetitive transcranial direct current stimulation; a-tDCS: anodal transcranial direct current stimulation; c-tDCS: cathodal transcranial direct current stimulation; C57BL/6: mouse strain; SC: stereotaxic coordinates; NDM: no disease model; SHR: spontaneous hypertensive rats; WKY: Wistar Kyoto rats; PFC: prefrontal cortex; mPFC: medial prefrontal cortex; dPFC: dorsolateral prefrontal cortex; DG: dentate gyrus; STR: *striatum*; S1: somatosensory cortex; V1: primary visual cortex; ITC: inferotemporal cortex; CA1: cornu ammonis 1 region in the hippocampus; TBI: traumatic brain injury; PRP: perampanel; CREB: cAMP response element-binding protein (transcription factor); CREB/CBP: cAMP response element binding protein; BDNF: brain-derived neurotrophic factor; DA: dopamine; GAP-43: growth associate protein 43; CaMKII: Ca^2+^/calmodulin-dependent protein kinase; mRNA: messenger ribonucleic acid; IP3R2: inositol triphosphate type 2 receptor; A1AR: adenosine A2A receptor; AMPAR: *α*-amino-3-hydroxy-5-methyl-4-isoxazolepropionic acid receptor; GluA1: AMPA receptor subunit A1; LFP: local field potential; LTP: long-term potentiation; PPF: paired pulse facilitation; EEG: eletroencephalography; A/m^2^: ampere per square meter; mA: milliampere; cm^2^: square centimeter; mm: millimeter; h: hour; min: minute; vs.: versus; Y: yes; N: no; N/A: not available.
